# Exploring the effect of use contexts on user engagement toward tourism short video platforms

**DOI:** 10.3389/fpsyg.2022.1050214

**Published:** 2022-11-24

**Authors:** Fei Qu, Nian Wang, Xianyan Zhang, Lan Wang

**Affiliations:** Business School, Guilin University of Technology, Guilin, China

**Keywords:** use contexts, user engagement, self-system model of motivational development, attitudinal engagement, behavioral engagement, short video

## Abstract

With tourism short video platforms’ increasingly fierce competition, retaining existing users and improving engagement has taken on greater theoretical and practical significance. Based on the self-system model of motivational development, this study, involving an empirical analysis of 252 user data samples, establishes a research model to determine how the use contexts affect users’ psychological process and finally lead to behavioral engagement. In particular, four use contexts of tourism short video platforms are proposed—namely, information acquisition, leisure and entertainment, attention obtainment, and social interaction. Different use contexts differ in the degree to which they satisfy users’ three psychological needs, thus influence their attitudinal engagement and behavioral engagement. The research results can provide theoretical and practical references on how to improve user engagement toward tourism short video platforms.

## Introduction

The 5G era of ultra-high speed, huge capacity, and low latency has brought new mobile technology advantages and flow empowerment, which have promoted the mobile short video platforms into becoming the primary means of information expression in modern citizen’s life (Yang and Bradford Brown, 2015). According to the China Internet Network Information Center’s most recent survey data, 888 million people in China had watched short videos as of June 2021, an increase of 14.4 million from 2020, with an average 125 min spent viewing short videos per person each day, while 53.5% of short video users browsed short video content every day (China Internet Network Information Center, 2021). Moreover, the mobile short video platforms represented by Tik Tok and Kwai have contributed to most time and flow increases in mobile Internet use, as they are the fundamental form of user expression and content consumption. At the same time, travel information search on short video platforms is becoming popular and rapidly growing into a mainstream marketing pattern of tourism, with benefits such as a large audience, strong interactivity, rich expressiveness, and low marketing costs. It not only shows the tourist sites to the public on line, but also promotes the sharing of travel-related experiences among users, who are drawn in by travel short video content. Therefore, searching for popular tourism topics on short video platforms has become a crucial factor in decision-making for potential tourists. Based on this, major short video platforms have developed strategies to encourage user participation, such as the “Support Plan” developed by Tik Tok and the “Guanghe Creators Conference” developed by Kwai. Tencent, one of the three giants among Internet companies in China, also actively promoted the “Wechat Channels” in 2020 to attract users to short videos as a source of social interaction. Baidu, on the other hand, has worked to add competitive content to its search engine, such as the development of short video searches.

In addition, the emergence of travel short videos has significantly shifted the role of users from passive content observers to active participants (Lee et al., 2018), who, themselves, become producers and creators of content through the sharing and communication of online short videos (Dolan et al., 2019). For example, many users share their travel experiences on short video platform, which attracts other users to the place and drives the emergence of many online popular tourist destinations. At the same time, users with travel intentions search for the interested destinations in advance on short video platforms to browse relevant comment or interact with each other about the destinations.

Therefore, major tourism short video platforms formulated various marketing strategies to attract large numbers of users; however, retaining these users is challenging. Typical platform users may not be loyal, which is not conducive to the platforms’ profit acquisition and long-term sustainability. At the same time, highly engaged users consistently share information and create content, which gives users more power, lower costs, and better coverage (Venkatesan, 2017). The key to the operation of travel short videos, then, is to enhance the user engagement with the platforms. Therefore, from the perspective of users’ psychological needs, this study explores the use contexts on user engagement to provide practical guidance for user retention and long-term engagement toward tourism short video platforms.

This research contributes to the following three aspects of this field. First, a research model of “use contexts-psychological process-behavioral outcomes” is developed to reveal the effect of use contexts on user engagement toward tourism short video platforms. Second, this study portrays the dynamic psychological transition process of user engagement in the context of tourism short video platforms, which makes the engagement process complete. Third, four use contexts are proposed—namely information acquisition, leisure and entertainment, attention obtainment, and social interaction, which provides new insights to explore the use contexts of tourism short video platforms.

## Theoretical background and literature review

### Self-system model of motivational development

According to the self-system model of motivational development (SSMMD), social contextual factors influence individuals’ self-systems to promote or weaken their engagement (Connell and Wellborn, 1991; Skinner et al., 2008). SSMMD is mainly based on self-determination theory (Deci and Ryan, 1985), which suggests that autonomy, competence, and relatedness are the three fundamental psychological demands of individuals. Autonomy refers to the need for people to believe that they can choose their own behavior, and when this need is satisfied, people can experience individual freedom; competence is the need for people to accomplish difficult and challenging tasks to attain desired results, and when this need is met, people feel a sense of mastery, achievement, and control. When people’s demand for mutual respect and connection with others is met and they receive social support from others, their need for relatedness is met. The self-system operation process is organized according to these three fundamental psychological needs. The SSMMD model suggests that only when the three psychological needs are satisfied can individuals develop in a positive direction, and then engagement can be occurred; otherwise, they may feel dissatisfied or depressed (Connell and Wellborn, 1991). The research model of this study is proposed based on the SSMMD. To be specific, social contextual factors correspond to the four use contexts of tourism short video platforms, which lead to three psychological needs for autonomy, competence, and relatedness of the users. As to SSMMD, when psychological needs are met, user engagement can be generated, including attitudinal engagement and behavioral engagement.

### Definition of user engagement

Most of the existing research on user engagement has focused on customer engagement, which has been studied in many fields, such as psychology, education, and most commonly, marketing. However, a unified understanding of the constituent dimensions of customer engagement in marketing is still lacking. Some researchers believe customer engagement is a one-dimensional concept that focuses on the psychological or behavioral dimension. The definition that focuses on the psychological dimension argues that customer engagement is a psychological state of motivation related to a particular company or brand and context-determined, generated from interaction with the focal object (e.g., company, brand, or platform; Bowden, 2009; Hollebeek, 2011). The definition that emphasizes behavior views customer engagement as a customer’s conduct toward a business or brand that extends beyond actual purchases, manifesting as word-of-mouth, helping others, providing feedback and suggestions, and so on (Van Doorn et al., 2010; Jaakkola and Alexander, 2014). Most scholars believe customer engagement is multidimensional, encompassing the three dimensions of cognition, emotion, and behavior (Brodie et al., 2011; Hollebeek et al., 2014). Cognition and emotion relate to the psychological manifestations of customer engagement, specifically, the psychological state of customers when they interact well with the focal object and co-create a customer experience, while the behavioral dimension focuses more on customers’ behaviors beyond making purchases (Hollebeek, 2011; So et al., 2016). This led some researchers to separate consumer engagement into psychological and behavioral dimensions, which they referred to as attitudinal engagement and behavioral engagement (Pansari and Kumar, 2016; Prentice et al., 2019). Attitudinal engagement refers to a psychological state of engagement that is expressed through two channels: cognition and emotion. Behavioral engagement, on the other hand, mostly manifests in customer behaviors, such as word-of-mouth, giving feedback, and so on.

According to the definition and dimensional division of customer engagement prevalent in the field of marketing, the study defines user engagement with tourism short video platforms as a positive relationship that users have established with the relevant platforms, which is reflected in the two dimensions of attitudinal engagement and behavioral engagement. Here, attitudinal engagement indicates a user’s psychological state of engagement with a platform, that is, a strong sense of belonging to and connection with the platform. Besides, attitudinal engagement is the positive emotional experience of users toward a tourism short video platform. When users are in positive emotion and connection with a platform, they will actively filter the negative evaluation and become more attached to the platform. Behavioral engagement is basically consistent with the prior studies concerning behavioral engagement of social media, referring to the users’ behaviors beyond benefits after interacting with the platform, which is reflected in six forms of engaged behaviors: viewing, reading comments, uploading, sharing, posting comments and liking (Khan, 2017). In this study, these six engaged behaviors were grouped into two categories, namely continued browsing and UGC (User-Generated Content). Continued browsing is a passive behavior whereby users frequently view short videos and read comments on the platform and gain the most value without actually engaging in any platform activities. By contrast, UGC is an active behavior whereby users actively participate in the activities on the platform and generate content, such as uploading and posting short videos, sharing short videos with others, commenting and liking their favorite short videos, and so on.

### Research review on user engagement

Many researchers have studied the user engagement or customer engagement with social media. The existing literature mainly discusses engagement from four perspectives: environmental level, organizational level, individual level, and individual-organizational interactive level. Researchers argue that factors at the environmental level associated with social media, such as virtual customer environmental characteristics (Verhagen et al., 2015), the digital experience environment (Rasool et al., 2020), and online service scenarios (De Oliveira Santini et al., 2020), positively impact user engagement. At the organizational level, the quality of community e-service (Pansari and Kumar, 2016), the characteristics of online brand community (Ul Islam and Rahman, 2017), and other factors can improve users’ engaged behaviors, such as re-patronage, word-of-mouth, and co-creation with the community. At the level of individual, factors such as motivation for community participation (Yoo and Gretzel, 2008), perceived value (Xie et al., 2021), quality of self-generated content (Quoquab et al., 2015), and network centrality (Kowalewski and Ruschoff, 2019) can improve user engagement by increasing users’ identification and trust in the community. Finally, social interactions through online communities (Luo et al., 2019), live interactions (Xue et al., 2020), and corporate responses to online reviews (Sheng, 2019) can promote user engagement at the individual-organizational interactive level.

This study examines the research on user engagement at the individual level. A review of the literature on this subject revealed a dearth of research in two areas. First, prior studies primarily focused on user engagement with traditional social media but lacked detailed discussion on user engagement toward tourism short video platforms, which emerged in recent years. Thus, the user engagement from individual level needs to be studied in detail. Second, while some researchers have discussed the impact of users’ motivation, perceived value, and other factors on engagement at the individual level in previous literature, their studies neglected the psychological change process that transforms ordinary users into engaged users. Beckers et al. (2014) believed that if engagement can be achieved, the first step is users’ psychological transformation. However, previous research was limited to static psychological variables, such as cognitive and emotional aspects of user engagement, which made the formation of the psychological process of user engagement static. That did not adequately account for the dynamic psychological shift from non-engaged to engaged users.

## Research model and hypotheses

**Figure 1:**
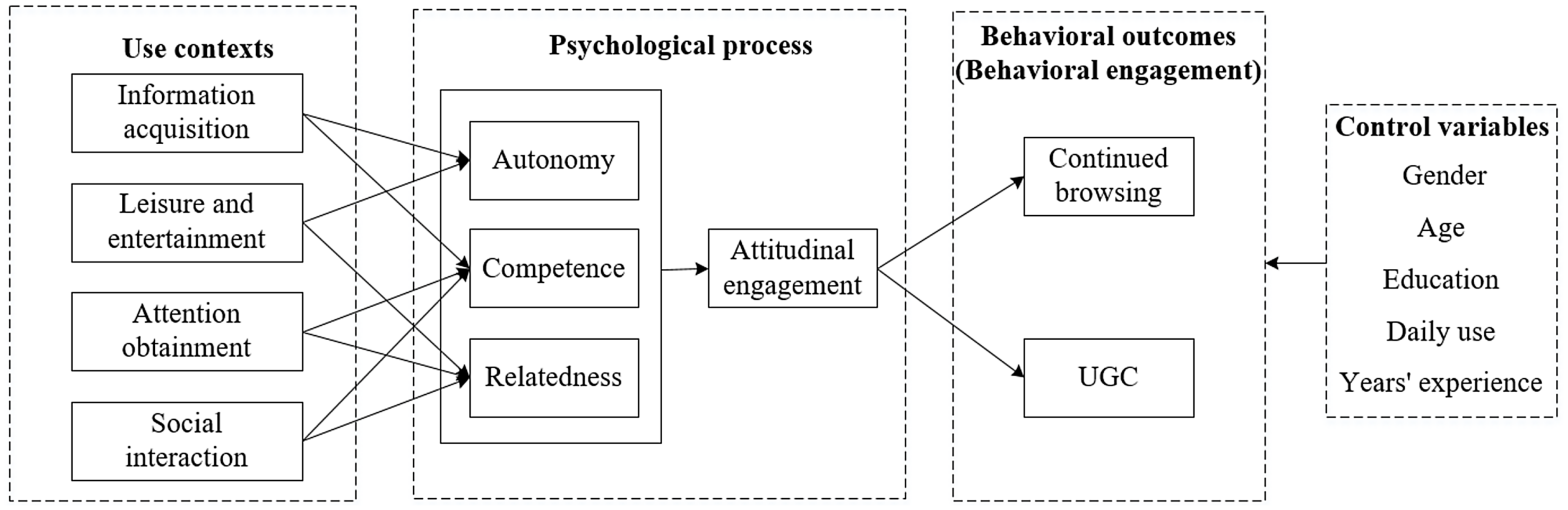
Research model for the current study.

Based on the SSMMD and the definition of user engagement, this study proposes a model for the effect of use contexts on user engagement from dynamic psychological perspective toward tourism short video platforms, as shown in Figure 1. First, users’ psychological needs for autonomy, competence, and relatedness are met based on four use contexts—namely, information acquisition, leisure and entertainment, attention obtainment, and social interaction—which, in turn, leads to users’ attitudinal engagement and behavioral engagement (including both continued browsing and UGC dimensions). The three psychological needs and attitudinal engagement are part of users’ psychological change process, while continued browsing and UGC are part of users’ behavioral outcomes. Gender, age, education, daily use, and years’ experience are all considered control variables.

### Use contexts of short video platforms

In this study, a grounded theory approach was used to explore the use contexts of tourism short video platforms. Following the basic logic of procedural grounded theory, a model of the use contexts for tourism short video platforms was obtained by following the steps of open coding, spindle coding, and selective coding. Firstly, to collect firsthand data, semi-structured interviews were conducted with 13 experienced users who often used travel short videos on the three platforms: Tik Tok, Kwai, and Bilibili. At the same time, a wide range of news reports, website information, and research literature related to the tourism short video platforms were collected as secondary data. Next, grounded theory was applied to encode the original primary and secondary data. By summarizing and refining each initial category, subcategory, and main category, the use contexts of tourism short video platforms were eventually determined, including the four dimensions of information acquisition, leisure and entertainment, attention obtainment, and social interaction. Specifically, information acquisition refers to the process of users obtaining relevant travel information to meet their needs by using tourism short video platforms. Leisure and entertainment is defined as the immersion, pleasure, and satisfaction that users gain from using travel short videos. Attention obtainment describes that users show themselves on the platforms to attract more attention and gain more recognition, thus boost their influence and popularity. Social interaction pertains to a special interpersonal relationship between users of tourism short video platforms.

### Research hypotheses

#### Use contexts and three psychological needs

Information acquisition describes the ways users obtain the travel information they need. This is the basic contextual factor for using travel short videos. Compared with text and pictures, short videos are more visually appealing (Lu et al., 2022). Accordingly, people are increasingly inclined to use short videos to quickly and effectively learn new travel information (Huangfu et al., 2022). The ability to freely access the information they need from short videos evokes a sense of freedom in users, which leads to a demand for autonomy. At the same time, users can effectively experience a sense of control and achievement in completing relevant tasks by acquiring knowledge for their own purposes from travel short videos. Hence, the following research hypotheses are proposed:

*H1a*: Information acquisition has a significant positive impact on autonomy.

*H1b*: Information acquisition has a significant positive impact on competence.

Secondly, the leisure and entertainment dimension pertains to people using travel short videos to relax themselves and gain pleasure. The literature documents that one of the most crucial reasons individuals use social media is for leisure and entertainment (Xu et al., 2012). In modern society, people work and study at an increasingly rapid pace, and as a result, many among them frequently experience depression. The emergence of travel short videos can help users to wind down from this fast-paced life and relax after work and school. Users gain a sense of freedom through tourism short video platforms, through which they can experience the local customs and cultural landscape of the world anytime and anywhere at a low cost. At the same time, users can also relax by following short videos of their favorite travel bloggers and interacting with them, which strengthens users’ connections and supports the need for relatedness. This leads to the following research hypotheses:

*H2a*: Leisure and entertainment have a significant positive impact on autonomy.

*H2b*: Leisure and entertainment have a significant positive impact on relatedness.

Thirdly, attention obtainment refers to users’ gaining attention from other users by uploading or sharing short travel videos, which, in turn, boosts their influence and popularity. For example, users make a 3–5 min short video of their own travel experiences and upload it to the platform: other users may watch it, comment on it, or like it. Producers can enhance their influence based on how many comments and likes the video received (Huangfu et al., 2022; Zhao and Shi, 2022). In this process, users gain attention and recognition from others by posting videos, interacting with other users, and liking videos, resulting in a sense of self-fulfillment, which satisfies their desire for competence. Gaining attention can help users communicate better, which satisfies the need for relatedness. This leads to the following research hypotheses:

*H3a*: Attention obtainment has a significant positive impact on competence.

*H3b*: Attention obtainment has a significant positive impact on relatedness.

Finally, social interaction refers to the friendship, kinship, and social support that people receive from other users through tourism short video platforms. Users exhibit their personalities and get to know one another in the process of using short videos. This facilitates travel information sharing and acquaintance of friends with common travel interests, and then establishes close, stable social ties (Bitrián et al., 2021). Moreover, users satisfy their need for relatedness in the process of interacting with other users; plus, they can follow each other’s friends. In addition, the social functions provided by travel short videos facilitate the exchange of information, helping users gain more knowledge and skills and further boosting their sense of achievement (Xi and Hamari, 2019). These factors motivate users and increase their desire to continuously improve their skills and progress to higher levels, while their need for competence is achieved in the process. As a result, the following research hypotheses are proposed:

*H4a*: Social interaction has a significant positive impact on competence.

*H4b*: Social interaction has a significant positive impact on relatedness.

#### Three psychological needs and attitudinal engagement

According to the SSMMD, engagement can occur when the needs for autonomy, competence, and relatedness are satisfied. When users experience a sense of freedom through independent choices in the platform, they can psychologically belong to, identify with and form an attitudinal engagement with the platform. They can also develop an attitudinal engagement with the platform when they feel a sense of self-achievement and control there. Finally, when users experience the pleasure of communication and interaction *via* the platform, the satisfaction of their need for relatedness promotes their attitudinal engagement with the platform. As a result, the following research hypotheses are proposed:

*H5a*: Satisfaction with autonomy has a significant positive impact on attitudinal engagement.

*H5b*: Satisfaction with competence has a significant positive impact on attitudinal engagement.

*H5c*: Satisfaction with relatedness has a significant positive impact on attitudinal engagement.

#### Attitudinal engagement and behavioral engagement

This study classifies users’ behavioral engagement with tourism short video platforms into two categories: continued browsing and UGC. When users have psychologically developed a strong sense of belonging to the platform—that is, when they have reached an attitudinal engagement with the platform—they behave in terms of continued browsing and UGC: in other words, they use the platform to view numerous short videos, post original videos, and share others’ videos, among other functions. As a result, the final two research hypotheses are proposed:

*H6a*: Attitudinal engagement has a significant positive impact on continued browsing.

*H6b*: Attitudinal engagement has a significant positive impact on UGC.

## Research design

### Data collection

In this study, the most popular mobile short video platforms in China—Tik Tok, Kwai and Bilibili—were chosen for investigation. Tik Tok, China’s leading destination for short-form mobile videos, empowers everyone to present a wide and diverse range of short video content on the platform. Kwai is a social network for short videos and trends, which aims to make it easier for people to record and share their lives. Bilibili is a full-spectrum video community with a diverse array of interests, as well as a story mode that provides users with short video content in their spare time. On these three platforms, there are independent travel short video sections, which involve many travel videos and ideas created by users. In addition, users can share travel experiences by interacting with others and browse destination-related videos and comments to make their travel plans colorful. User engagement with the platforms is steadily developed during these activities. Therefore, the survey was conducted among users who often used travel short videos on the three platforms. The data were collected *via* a questionnaire that consisted of two parts. The first part was designed to gather personal statistical information such as gender, age, education, daily use and years’ experience. In terms of the variables, each measurement item in the second part was presented using a five-point Likert scale ranging from “extremely inconsistent” to “very consistent.” After designing the initial questionnaire, the research group conducted a pre-survey to revise the wording of any items that were ambiguous or not clearly described. The pre-survey also allowed for any junk items with low reliability to be removed from the questionnaire. The final questionnaires were distributed *via* the Questionnaire Star service platform from November 2021 to December 2021. A total of 379 questionnaires were collected. After excluding any invalid questionnaires, 252 valid questionnaires were obtained, which represented an effective response rate of 66.5%. The specific structure of the valid sample is shown in Table 1. The majority of the sample were female, were aged 18–30 years, had an undergraduate education, used travel short videos more than five times each day, and had used short videos for 2 to 3 years. Moreover, Tik Tok and Bilibili were the two most popular short video platforms.

**Table 1:** Description of the valid sample structure.

Characteristics	Options	Proportion
Most frequently used short video platforms	Tik Tok	62.7%
	Bilibili	20.6%
	Kwai	7.5%
	WeChat channels	2.0%
	MicroBlog channels	1.6%
	Others	5.6%
Gender	Male	33.3%
	Female	66.7%
Age	Under 18 years old	0.4%
	18–30 years old	82.1%
	31–40 years old	13.1%
	41–50 years old	3.6%
	51 years and older	0.8%
Education	High school or below	5.2%
	Tertiary students	4.8%
	Undergraduates	71.3%
	Master’s and above	18.7%
Daily use	Within 1 time	4.4%
	1–2 times	25.4%
	3–4 times	32.1%
	5 times or more	38.1%
Years’ experience	Less than 1 year	5.2%
	1–2 years	25.0%
	2–3 years	38.5%
	More than 3 years	31.3%

### Measurement of variables

To ensure the reliability and validity of this study’s scale, the measurement items for the research variables were referred to established mature scales. The foreign scales were translated into English and Chinese in a double-blind format, and the measurement items of the scales were appropriately modified according to the Chinese context. Among them, the scale proposed by Dolan et al. (2016) was used for information acquisition, with a total of four items, such as “travel short videos provide me with travel information and knowledge I am interested in.” Leisure and entertainment were measured using the scale provided by Park et al. (2009), with three items, including “browsing travel short videos is relaxing.” Attention obtainment was measured with four items from the scale developed by Muhammad et al. (2021), such as “sharing my interests and travel experiences with others through travel short videos to improve my profile.” Raacke and Bonds-Raacke (2008) scale was adopted for social interaction, measured with four items, such as “travel short videos help me get to know more people with similar interests.” Autonomy was measured using Xi and Hamari (2019) scale, which consisted of four items, like “I am free to decide what to do in travel short videos.” Competence was based on Johnston and Finney (2010) scale with three items, such as “I feel that I am capable of using travel short videos to learn interesting new knowledge or skills.” Relatedness was also based on Xi and Hamari (2019) scale; this variable was measured with four items, including “I feel supported by others when using the travel short videos,” while attitudinal engagement was measured using Vivek (2009) scale with six items, such as “I am enthusiastic about participating in travel short videos.” Continued browsing was assessed using Papacharissi and Rubin (2000) scale with three items, one of which was “I often search for travel short videos I am interested in on this short video platform.” Finally, UGC was evaluated using four items from the scale developed by Wang and Zhang (2018), such as “I often record and post my travel experiences and suggestions on this short video platform.”

## Data analysis and model testing

### Reliability and validity tests

#### Reliability test

In the 252 valid questionnaires, Cronbach’s α for all the variables tested for reliability except information acquisition was above 0.7, while Cronbach’s α for information acquisition was slightly below 0.7, as shown in Table 2. In general, the scale had good reliability.

**Table 2:** Results of reliability and validity tests.

Variables	Items	Standardized factor loadings	Cronbach’s α	CR	AVE
Information acquisition	IA1	0.687	0.649	0.795	0.493
	IA2	0.738			
	IA3	0.740			
	IA4	0.638			
Leisure and entertainment	LE1	0.860	0.840	0.904	0.758
	LE2	0.888			
	LE3	0.863			
Attention obtainment	AO1	0.778	0.927	0.926	0.758
	AO2	0.892			
	AO3	0.896			
	AO4	0.911			
Social interaction	SI1	0.802	0.875	0.914	0.727
	SI2	0.861			
	SI3	0.882			
	SI4	0.864			
Autonomy	AUT1	0.757	0.786	0.865	0.617
	AUT2	0.842			
	AUT3	0.740			
	AUT4	0.798			
Competence	COM1	0.807	0.789	0.859	0.670
	COM2	0.856			
	COM3	0.791			
Relatedness	REL1	0.839	0.891	0.925	0.754
	REL2	0.880			
	REL3	0.868			
	REL4	0.885			
Attitudinal engagement	AE1	0.772	0.875	0.907	0.618
	AE2	0.823			
	AE3	0.795			
	AE4	0.813			
	AE5	0.791			
	AE6	0.719			
Continued browsing	CB1	0.771	0.700	0.832	0.623
	CB2	0.833			
	CB3	0.762			
UGC	UGC1	0.754	0.783	0.861	0.609
	UGC2	0.796			
	UGC3	0.717			
	UGC4	0.848			

#### Validity test

In this study, convergent validity was confirmed using standardized factor loadings, composite reliability (CR), and average variance extracted (AVE). According to the results as presented in Table 2, each standardized factor loading exceeded the required 0.5, the AVE of each variable also basically exceeded the acceptable level of 0.5, and the CR was greater than 0.7, indicating that the scale had good convergent validity. In addition, the square roots of the AVE values of all variables were greater than their correlation coefficients with other variables, indicating good discriminant validity for the scale.

### Model testing and result analysis

In this study, Mplus 7.0 was used to test the research hypotheses. First, the variables were centered separately to avoid the problem of multicollinearity. Next, the research hypotheses proposed in this paper were tested; the results are shown in Table 3. The model fit index were χ2/df = 1.839, CFI = 0.879, TLI = 0.870, RMSEA = 0.058, and SRMR = 0.073.

**Table 3:** Test results of the research model.

Independent variables	Dependent variable 1	Dependent variable 2	Dependent variable 3	Dependent variable 4	Dependent variable 5	Dependent variable 6
	Autonomy	Competence	Relatedness	Attitudinal engagement	Continued browsing	UGC
**Main effects**						
Information acquisition	0.504***	0.372***	—	—	—	—
Leisure and entertainment	0.431**	—	0.030	—	—	—
Attention obtainment	—	0.363***	0.471***	—	—	—
Social interaction	—	0.339***	0.382***	—	—	—
Autonomy	—	—	—	0.460***	—	—
Competence	—	—	—	0.504***	—	—
Relatedness	—	—	—	0.131	—	—
Attitudinal engagement	—	—	—	—	0.806***	0.772***
**Control variables**						
Gender	—	—	—	—	−0.073	0.049
Age	—	—	—	—	−0.105	0.026
Education	—	—	—	—	−0.103	0.059
Daily use	—	—	—	—	0.032	0.037
Years’ experience	—	—	—	—	0.069	−0.135*
**Fit index**	χ^2^/ df = 1.839, CFI = 0.879, TLI = 0.870, RMSEA = 0.058, SRMR = 0.073					

*p < 0.05, **p < 0.01, ***p < 0.001.

Based on the test results for the main effects, information acquisition had a significant effect on autonomy (*β* = 0.504, *p* < 0.001) and competence (*β* = 0.372, *p* < 0.001), thereby confirming hypotheses H1a and H1b. Leisure and entertainment had a significant effect on autonomy (*β* = 0.431, *p* < 0.01), to hypothesis H2a was confirmed, but it did not reach a statistically significant level on relatedness (*β* = 0.030, *p* > 0.05), so hypothesis H2b was not confirmed. Attention obtainment had a significant effect on competence (*β* = 0.363, *p* < 0.001) and relatedness (*β* = 0.471, *p* < 0.001), as such, hypotheses H3a and H3b were confirmed. Moreover, social interaction had a significant effect on competence (*β* = 0.339, *p* < 0.001) and relatedness (*β* = 0.382, *p* < 0.001), indicating hypotheses H4a and H4b were confirmed. Autonomy (*β* = 0.460, *p* < 0.001) and competence (*β* = 0.504, *p* < 0.001) had a significant effect on attitudinal engagement, which verified H5a and H5b, while relatedness did not have a statistically significant effect on attitudinal engagement (*β* = 0.131, *p* > 0.05), so hypothesis H5c was not verified. Attitudinal engagement had a significant effect on continued browsing (*β* = 0.806, *p* < 0.001) and UGC (*β* = 0.772, *p* < 0.001), so H6a and H6b were validated. Five control variables were selected for this study, of which gender, age, education, and daily use had no significant effect on either continued browsing or UGC; years’ experience had no significant effect on continued browsing and only a slight effect on UGC (*β* = −0.135, *p* < 0.05).

## Research conclusion and discussion

### Research conclusion

This study proposes four use contexts of tourism short video platforms—namely, information acquisition, leisure and entertainment, attention obtainment, and social interaction—related to meeting users’ three fundamental psychological needs relevant to improving their attitudinal and behavioral engagement with tourism short video platforms. The main findings of the study follow.

First, the four use contexts associated with tourism short video platforms were found to have positive effects on three psychological needs. Consistent with the hypotheses that informed this study, information acquisition satisfied users’ autonomy and competence, while leisure and entertainment satisfied users’ autonomy, and attention obtainment and social interaction both satisfied users’ competence and relatedness. In contrast to the hypotheses, however, leisure and entertainment did not have a significant effect on users’ relatedness. One possible explanation for this result is that users use tourism short video platforms for leisure and entertainment purposes, mainly to relax and enjoy the sense of freedom and pleasure that such platforms bring to them. This state of self-relaxation does not foster a sense of relatedness on the part of users in terms of making friends or interacting with current friends.

Second, autonomy and competence were observed to have significant impacts on users’ attitudinal engagement. More specifically, the results showed that when users acquired autonomy and competence through their use of the tourism short video platforms, they were able to achieve attitudinal engagement with the platforms. Yet, inconsistent with the findings of previous studies, relatedness did not have a significant effect on users’ attitudinal engagement. This difference is most likely due to the fact that prior studies were conducted based on the social media context, which placed a strong emphasis on social factors and so prompted users to exhibit strong relatedness. Thus, the satisfaction of relatedness can result in users’ attitudinal engagement with social platforms. Tourism short video platforms differ from traditional social media sites in that their social function is relatively weak while self-presentation function is extremely strong. Even if users can obtain relatedness through their use of tourism short video platforms, this relatedness does not have a significant impact on their attitudinal engagement because of weak social function.

Third, attitudinal engagement was shown to have a significant effect on users’ behavioral engagement. In accordance with previous findings, this study determined that users’ attitudinal engagement significantly influenced their behavioral engagement. When users experience psychological attitudinal engagement, they are more likely to continue using the platform to view various travel short videos. At the same time, more UGC behaviors (e.g., posting, commenting, sharing and liking) are also more likely to be displayed by users.

Fourth, this study found that the older the user, the more inclined they were to UGC on the platform, while the younger the user, the more willing they were to continuously browse on the platform. One possible explanation for this age-related difference is that younger people have more stressful work and study and so are more willing to continuously view travel short videos on the platform during their free time, whereas older users have relatively more time to spend on making, posting and commenting on travel short videos. Similarly, the more educated users tended to be more inclined toward UGC, while the less educated users were more willing to browse videos on the platform. This may be because both the production and posting of travel short videos require the learning and mastering of certain information technology skills that users with higher education levels are likely to have more knowledge and experience of, which means they are more comfortable with video production than users with lower education levels. Therefore, users with higher education levels typically exhibit more UGC behaviors. Furthermore, users who had been using short video platforms for a longer period of time were more inclined to keep viewing videos rather than generating UGC behaviors when compared with those who had spent a shorter period of time using tourism short video platforms. This finding is very interesting and suggests that established users may be lazier, while newer users may be relatively more diligent and willing to post and comment on travel short videos.

### Theoretical contributions

First, the existing literature focuses on the user engagement of traditional social media, while lacking discussion on the user engagement for the current popular tourism short video platforms. Based on the SSMMD, this paper constructs an analytical framework of “use contexts-psychological process-behavioral outcomes” to reveal the effect of use contexts on user engagement toward tourism short video platforms.

Second, previous studies have not paid sufficient attention to users’ dynamic psychological change process when examining user engagement. This study refines the psychological process of user engagement in the context of travel short videos, breaking it down into two stages: psychological need and attitudinal engagement. The refined psychological process portrays the dynamic transition from three psychological needs to attitudinal engagement, making the psychological process of user engagement complete and no longer limited to a single, static psychological factor.

Third, the existing literature lacks relevant studies on use contexts of tourism short video platforms. In this paper, four use contexts of tourism short video platforms are proposed—namely information acquisition, leisure and entertainment, attention obtainment, and social interaction. The findings reveal that different use contexts differ in the degree to which they satisfy users’ psychological needs, thus influencing their attitudinal engagement and behavioral engagement. Therefore, this paper provides new insights to explore the use contexts of tourism short video platforms.

### Management insights

As competition between tourism short video platforms becomes increasingly fierce, the question of how to influence users to form a long-term engagement with the platform has become an important reality for these platforms. The findings of this paper provide two practical insights into how tourism short video platforms can retain engagement users.

First, tourism short video platforms need to pay attention to the four use contexts. The findings of this paper show that the four use contextual factors of information acquisition, leisure and entertainment, attention obtainment, and social interaction have different degrees of influence on each of three psychological needs. Therefore, platforms should fully consider addressing the use contextual factors of different users. For example, some people use tourism short video platforms mainly for information acquisition; in this case, the platform needs to provide or recommend valuable travel information for different users through the big data recommendation mechanism to meet their information needs. Many users rely on tourism short video platforms for leisure and entertainment. For these users, the platform needs to post or encourage other users to upload as many interesting and exciting travel videos as possible so they can find relaxation through the platform. Some users turn to the tourism short video platforms to gain attention: the platform needs to provide incentives, such as points, badges, or leaderboards, to help them achieve higher levels of success through their efforts. In addition, platforms need to provide users with as many opportunities as possible to socially engage with strangers, acquaintances, or friends, for example, by allowing them to share their own pre-tour preparations or post-tour advice, and so on. At the same time, they are encouraged to invite their friends to join the platform to further enhance their social interactions.

Second, tourism short video platforms should fully meet users’ basic psychological needs to promote behavioral engagement. Research findings show that when platforms meet individual users’ needs for autonomy and competence, they can achieve significant attitudinal engagement, which, in turn, influences their continued browsing and UGC. Therefore, tourism short video platforms should create a favorable use environment to fully satisfy users’ needs for autonomy and competence. Various specific strategies can be employed. First, while providing users with substantial support, tourism short video platforms should open certain permissions to strength their autonomy. Active users should be given appropriate authorization to participate in activities, such as reviewing works and initiating topics, to enhance their unique experience on the platform and increase their freedom. In addition, the platform can encourage users to participate in the construction of their travel videos to strengthen their active innovation and creative spirit. For example, users are allowed to adjust shooting parameters and even upload their favorite filters, special effects, and music. Second, tourism short video platforms can screen user behaviors through methods such as big data tracking, which can adopt multiple incentive strategies targeting different types of users to improve their competence. For example, platforms should give users who share and interact more often increased visual recognition and priority recommendations on their video works, and they should establish a badge system to satisfy users’ sense of achievement and motivate them to grow themselves. For users whose works receive a high degree of attention, platforms should give certain material incentives to meet the user’s need to be recognized. In addition, for content such as excellent video works and comments, the tourism short video platforms can set up star tags to draw the attention of other users, which not only enhances the exposure of excellent content but also inspires other users.

### Limitations and future research

This study has some limitations that can provide promising directions for future research. First, while the inclusion of control variables can alleviate endogeneity problems in the model (Bitrián et al., 2021), it is not possible to completely eliminate the endogeneity. Among them, years’ experience slightly and negatively influences UGC, indicating that years’ experience has a slight impact on the dependent variable. Therefore, this is one of the limitations of this study. Second, this study employed a one-off questionnaire to collect data. Future research can try to obtain objective secondary data from short video platforms and adopt different methods to further corroborate the validity of the model. Third, this paper mainly selects tourist users from short video platforms such as Tik Tok and Kwai, which are currently popular in China, as the objects for research; future research can consider selecting short video platforms in other countries, such as YouTube Shorts and Instagram Reels, for comparative research to test the differences and similarities of relevant findings among China and other countries. Finally, the research model only considers the effect of use contexts. Other important factors that affect user engagement have not been included in the research model, such as the personality traits of users and the characteristics of the short video platforms, which can be considered in future research.

## Data availability statement

The original contributions presented in the study are included in the article/supplementary material, further inquiries can be directed to the corresponding author.

## Ethics statement

Ethical review and approval were not required for the study on human participants in accordance with the local legislation and institutional requirements. Written informed consent for participation was not required for this study in accordance with the national legislation and the institutional requirements.

## Author contributions

FQ: conceptualization, methodology, and writing—review and editing. NW: investigation and writing—original draft. XZ: supervision and writing—review and editing. LW: data analysis. All authors contributed to the article and approved the submitted version.

## Funding

This research was supported by grant from the National Natural Science Foundation of China and Natural Science Foundation of Guangxi (72062011, 2018GXNSFBA281004, 71962005, AD19245134).

## Conflict of interest

The authors declare that the research was conducted in the absence of any commercial or financial relationships that could be construed as a potential conflict of interest.

## Publisher’s note

All claims expressed in this article are solely those of the authors and do not necessarily represent those of their affiliated organizations, or those of the publisher, the editors and the reviewers. Any product that may be evaluated in this article, or claim that may be made by its manufacturer, is not guaranteed or endorsed by the publisher.
